# Ontario Veterinary College

**Published:** 1901-04

**Authors:** 


### COMMENCEMENT EXERCISES.

### ONTARIO VETERINARY COLLEGE.

The closing exercises of the session of 1900–1901 of the Ontario Veterinary College were held in the college building on March 27th. The principal, Prof. A. Smith, F.R.C.V.S., took the chair, and with him on the platform were: Mr. A. Pattullo, M.P.P.; Prof. Baker, Toronto University; Prof. Mavor, Toronto University; Mr. Hill, Industrial Exhibition, Toronto; Mr. H. S. Wende, V.S., President Ontario Veterinary Association, and Dr. Duncan, M.D. Prof. A. Smith opened the meeting with a short address, and called on Dr. Duncan to read the graduating and honor lists, also the list of prize-winners.

Mr. Pattullo said he was astonished on looking over the list of graduates at the large number who came from all parts of the continent of North America. He could remember when the old-time practitioner held a far different position in the view of the public to the college graduates of to-day. Now the veterinary graduate is, or should be, on a social equality with anyone. The attention of the British Government had been called to Canada as a suitable field in which remounts may be procured for army purposes. A country that can produce good men can produce good horses, and he looked upon the veterinary profession as a most important aid in encouraging and improving the breeding of a good and suitable class of horses. There is a splendid future in Canada for the production of a good class. He believed that the prospects of the profession were never so good as now, and will still keep improving. In conclusion, he was much pleased to see present so many young men from the United States, and he trusted and believed that the peace and good-will that existed between ourselves and our brethren across the borders would long continue. He wished success to the graduates in their future career, wherever they may be located.

The other guests also addressed the audience.

The following is the list of graduates :

Hal. L. Bellinger, Hickory Corners, Mich.; G. Elmer Bitgood, Voluntown, Conn.; William A. Boucher, Minneapolis, Minn.; Francis W. Buckle, Guelph ; Hiram Burlingham, Wellington ; Thomas Bryant, Wayland, Mass.

William A. Connoly, Fullerton, Cal.; George T. Crowley, New Britain, Conn.; Albert B. Culley, Avon, N. Y.

Frederick A. Davis, Dunstable, Mass.; C. E. Dickerman, Montpelier, Vt.; George B. Duncan, Beloit, Kansas.

O. H. Eliason, Scandinavia, Wis.; Charles H. Epps, Richmond, Va.; Claude C. Evely, St. Thomas, Ont.; R. Frank Erwin, Pinckney, Mich.

Fred. D. Fordham, Watkins, N. Y.; William D. Forsythe, Southbridge, Mass.; Thomas Fraser, Richmond, Va.; George L. Frese, Elmore, Ohio.

Robert G. George, Piqua, Ohio ; Walter C. Giller, Rood House, Ill.; Nathaniel S. Glass, Chesley.

Charles E. Howard, Leonardsville, N. Y.

Percy S. Isaacson, Hardingham, Norfolk, England.

Matthew S. Kennedy, Carman, Man.; William J. Kirk, Sharon, Pa.

William M. Lowery, Cliton.

John P. McCoy, Minden City, Mich.; William McDonald, Florence ; George A. McLevey, Florence.

C. Arthur Mack, Carberry, Man.; Samuel M. Minzer, Wilmot, Ohio ; James J. Murison, Cannington Manor, Assa.; Thomas H. Monahan, Providence, R. I.

William J. Pedden, Parkhill, Ont.; John Perschbacher, Grand Rapids, Mich.; T. Milton Pine, Allisonville, Ont.; L. J. Price, Liberty Centre, Ohio ; P. J. Purcell, Bradford, Pa.

J. C. Rasmussen, Tampico, Ill.; Clarence Ransford Richards, Victoria, B. C.; T. Herbert Richards, Beaumaris, Ont.; A. L. Ramage, Calgary, N. W. T.

Zen. W. Seibert, Mansfield, Ohio ; John Thomas Sharpe, Carman, Man.; Robert James Shine, Brussels, Ont.; Summer S. Smiley, Carman, Man.; William F. Smiley, Carman, Man.; B. E. Springer, Akron, Ohio ; John F. Sylvester, Carman, Man.; F. F. Sheets, Van Wert, Ohio.

R. Claude Titus, Hillier, Ont.; F. H. Tucker, Lincoln, Neb. Carr R. Webber, Rochester, N. Y.; Leslie Willoughby, Elmwood, Ont.; George Wooldridge, Lowell, Ind.

PRIZES AND HONORS. *Diseases and Treatment* (*Seniors*). First prize—C. R. Richards (silver medal). Second prize—L. Willoughby. Third prize—J. P. McCoy.

HONORS. G. Bitgood, W. Boucher, F. Buckle, T. Bryant, W. Connoly, G. Crowley, A. B. Cully, G. B. Duncan, O. H. Eliason, C. C. Evely, W. D. Forsythe, T. Fraser, F. D. Fordham, G. Frese, R. G. George, W. Giller, C. E. Howard, P. Isaacson, W. J. Kirk, William Lowery, J. P. McCoy, G. A. McLevey, C. A. Mack, S. Mizer, T. Monahan, J. J. Murison, M. T. Pine, L. J. Price, P. J. Purcell, A. Ramage, C. R. Richards, T. H. Richards, F. Sheets, R. J. Shine, W. F. Smiley, B. E. Springer, F. H. Tucker, C. R. Webber, L. Willoughby.

*Materia Medica* (*Seniors*). First prize—Leslie Willoughby. Second prize—C. R. Richards. Third prize—C. C. Evely.

HONORS. T. Bryant, H. Bellinger, W. Boucher, G. Bitgood, W. Connoly, G. Crowley, A. B. Culley, G. B. Duncan, G. Frese, T. Fraser, F. Fordham, R. George, P. S. Isaacson, William Kirk, T. Monahan, J. J. Murison, S. Mizer, J. P. McCoy, R. Norton, W. J. Pedden, F. Pine, T. Ramage, W. F. Smiley, R. E. Springer, R. C. Titus, C. R. Webber.

*Chemistry* (*Seniors*). First prize—Thomas H. Monahan and Leslie Willoughby, equal. Second prize—B. E. Springer and R. E. George, equal.

HONORS. G. Bitgood, W. Boucher, H. Bellinger, F. Buckle, W. Connoly, A. B. Culley, C. E. Dickerman, C. C. Evely, G. F. Frese, Thomas Fraser, C. E. Howard, P. Isaacson, J. P. McCoy, A. E. Melhuish, C. A. Mack, J. J. Murison, R. J. Norton, A. L. Pine, C. R. Richards, J. C. Rasmussen, T. H. Richards, R. J. Shine, W. F. Smiley, C. R. Webber, G. Wooldridge.

*Pathology* (*Seniors*). First prize—Leslie Willoughby. Second prize—A. B. Culley. Third prize—C. R. Richards.

Honors. H. L. Bellinger, G. E. Bitgood, W. A. Boucher, F. Buckle, T. Bryant, W. A. Connoly, G. T. Crowley, C. H. Epps, C. C. Evely, F. Fordham, G. L. Frese, T. Fraser, R. G. George, W. Giller, C. Howard, P. Isaacson, M. S. Kennedy, J. P. McCoy, S. Mizer, J. J. Murison, T. Monahan, R. J. Norton, M. Pine, L. Price, A. L. Ramage, T. H. Richards, J. Sharpe, W. F. Smiley, B. E. Springer, R. C. Titus, F. H. Tucker, C. R. Webber, G. Wooldridge.

*Physiology* (*Seniors*). First prize—C. R. Richards. Second prize—Leslie Willoughby. Third prize—J. P. McCoy.

Honors. F. Buckle, G. Bitgood, W. A. Boucher, C. Evely, Thomas Fraser, P. Isaacson, M. S. Kennedy, Thomas Monahan, T. M. Pine, A. L. Ramage, J. C. Rasmussen, W. F. Smiley.

*Anatomy* (*Seniors*). First prize—C. R. Richards, silver medal. Second prize—Leslie Willoughby. Third prize—J. P. McCoy.

Honors. H. Bellinger, G. Bitgood, W. A. Boucher, F. Buckle, Thomas Bryant, W. A. Connoly, G. T. Crowley, A. B. Culley, C. C. Evely, Thomas Fraser, C. E. Howard, Percy Isaacson, M. S. Kennedy, J. L. McCoy, J. J. Murison, A. E. Melhuish, T. H. Monahan, R. J. Norton, T. H. Richards, J. C. Rasmussen, A. L. Ramage, R. J. Shine, B. E. Springer, R. C. Titus, George Woolridge, Milton Pine.

*Entozoa* (*Seniors*). First prize—P. S. Isaacson and C. R. Richards, equal.

Honors. W. Boucher, G. Bitgood, Thomas Bryant, H. Bellinger, F. W. Buckle, G. T. Crowley, A. B. Culley, W. A. Connoly, O. H. Eliason, W. D. Forsythe, W. C. Giller, M. S. Kennedy, J. P. McCoy, W. McDonald, A. E. Melhuish, J. J. Murison, L. J. Price, J. C. Rasmsusen, A. L. Ramage, B. E. Springer, W. F. Smiley, C. C. Webber, Leslie Willoughby.

*Dissected Specimens*. First prize—Leslie Willoughby, gold medal, given by the Toronto Industrial Association. Second prize—F. W. Buckle. Third prize—G. T. Crowley.

*Best General Examination*. First prize—C. R. Richards, gold medal, given by the Ontario Veterinary Association.

Honors. C. C. Evely, L. J. Price, Leslie Willoughby.

*Primary Examinations*. Anatomy—Alexander Doherty, Jr., J. Leonard Faragher, Albert T. Ford, John T. McCoy, Arthur E. Melhuish, Robert J. Norton, S. C. Neff, Jr., J. Arthur Royce, Arthur R. Torrie.

*Materia Medica*. Wilson A. Bisbee, Albert Henry Fehr, William D. McMullen, Arthur E. Melhuish, Robert J. Norton, S. C. Neff, Jr., J. Arthur Royce, Arthur R. Torrie.

*Diseases and Treatment* (*Juniors*). First prize—S. Ransom. Second prize—J. H. Pickering. Third prize—G. A. Harvey.

HONORS. F. Anderson, H. Armour, H. Berry, A. Blacklock, C. Bonsfield, J. P. Bridge, J. Burton, G. Candage, C. Chollar, M. G. Connolly, C. S. Cooper, J. S. Darling, F. Delaine, G. Dick, R. Dinnis, C. H. Doyle, E. Farewell, R. C. Freeman, E. R. Fryer, W. Gill, Gardiner Harvey, J. S. Hollingsworth, R. A. Hume, W. Irwin, J. W. Jackson, J. A. McLeish, E. J. Magee, M. Marshall, W. Mills, J. P. Molloy, W. J. Neil, H. Nobles, J. H. Poe, H. Pomfret, H. Rea, E E. Robbins, G. Schneider, O. Selle, J. Sheehan, B. C. Smith, L. Snider, C. Stevens, T. Stover, W. C. Van Allstyne, R. T. Williams.

*Anatomy* (*Juniors*). First prize—E. E. Robbins, silver medal. Second prize—W. C. Van Allstyne. Third prize—G. A. Dick.

HONORS. G. Candage, R. Dinnis, E. Farewell, G. Harvey, Gardiner Harvey, J. G. McLeish, C. E. Poe, J. H. Pickering, H. Pomfret, S. Ransom, O. O. Selle, T. J. Stover, C. C. Stevens, G. L. Schneider, R. T. Williams.

*Physiology* (*Juniors*). First prize—T. J. Stover, silver medal. Second prize—J. H. Pickering. Third prize—J. Hollingsworth.

HONORS. H. Armour, E. Roy Farewell, Geo. A. Harvey, Gardiner Harvey, J. P. Molloy, J. A. McLeish, H. Pomfret, E. E. Robbins, S. Ransom, B. C. Smith, G. S. Schneider.

*Chemistry* (*Juniors*). First prize—J. H. Pickering. Second prize—G. A. Harvey and S. Ransom, equal.

HONORS. H. Armour, G. Candage, T. Colling, F. Delaine, J. B. Darling, E. R. Farewell, R. E. Freeman, G. Harvey, R. Hume, J. S. Hollingsworth, E. J. Magee, J. P. Molloy, W. J. Neil, H. W. Nobles, H. Pomfret, E. Robbins, G. S. Schneider, B. C. Smith, O. Selle, C. C. Stevens, J. J. Stover, R. J. Williams.

*Pathology* (*Juniors*). First prize—S. Ransom. Second prize—G. A. Harvey. Third prize—T. J. Stover.

HONORS. F. Anderson, H. Berry, T. Blacklock, C. Bonsfield, P. Bridge, J. F. Colling, C. J. Cooper, J. S. Darling, F. J. Delaine, G. A. Dick, R. W. Dinnis, C. H. Doyle, E. R. Farewell, D. Fleming, R. E. Freeman, E. L. Fryer, W. Gill, G. Harvey, J. S. Hollingsworth, R. A. Hume, W. Irwin, J. A. McLeish, E. J. Magee, M. Marshall, W. L. Mills, J. P. Molloy, H. W. Nobles, W. J. Neil, J. H. Pickering, C. E. Poe, H. Pomfret, H. E. Rea, E. E. Robbins, G. L. Schneider, O. Selle, G. F. Sheehan, B. C. Smith, L. Snyder, C. Stevens, W. C. Van Allstyne, R. T. Williams.

